# Identifying distinct subgroups with severe pain in sickle cell disease: A cluster analysis of the GRNDaD multi-center registry

**DOI:** 10.1371/journal.pone.0320889

**Published:** 2025-05-15

**Authors:** Martha O. Kenney, Samuel Wilson, Morgan Rosser, Sophie Lanzkron, Julie Kanter, Susan Padrino, Payal Desai, Deepa Manwani, Alice Cohen, Stephanie Guarino, Ward Hagar, Joshua Field, Jane Little

**Affiliations:** 1 Department of Anesthesiology, Division of Pediatric Anesthesiology, Duke University Medical Center, Durham, North Carolina, United States of America; 2 Department of Medicine, Division of Hematology, University of North Carolina at Chapel Hill School of Medicine, Chapel Hill, North Carolina, United States of America; 3 UNC Blood Research Center, University of North Carolina at Chapel Hill, Chapel Hill, North Carolina, United States of America; 4 Department of Anesthesiology, Duke University Medical Center, Durham, North Carolina, United States of America; 5 Department of Medicine, Division of Hematology, Thomas Jefferson University, Philadelphia, Maryland, United States of America; 6 Division of Hematology and Oncology, University of Alabama, Birmingham, Alabama, United States of America; 7 School of Medicine, Case Western Reserve University School of Medicine, Cleveland, Ohio, United States of America; 8 Levin Cancer Institute, Atrium Health, Charlotte, North Carolina, United States of America; 9 Department of Pediatrics, Albert Einstein College of Medicine and the Children’s Hospital at Montefiore (CHAM), Bronx, New York, United States of America; 10 Newark Beth Israel Medical Center, Newark, New Jersey, United States of America; 11 Department of Medicine, ChristinaCare, Wilmington, Delaware, United States of America; 12 Department of Pediatrics, Nemours Children’s Health, Wilmington, Delaware, United States of America; 13 Department of Pediatrics, UCSF Benioff Children’s Hospital, Oakland, California, United States of America; 14 Department of Medicine, Medical College of Wisconsin, Milwaukee, Wisconsin, United States of America; Makerere University / Mulago National Referral Hospital, UGANDA

## Abstract

Sickle cell disease (SCD) affects millions of individuals worldwide, and is characterized by both acute, episodic pain and chronic, persistent pain. Despite the significant burden of the disease, clinicians continue to face significant challenges in treating SCD pain due to variability in pain experiences. The objectives of this study were (1) to identify distinct pain subgroups based on demographic and biopsychosocial characteristics and (2) to evaluate the relationship between the subgroups and pain impact – a SCD-disease specific measure of pain interference. To achieve these objectives, we performed a hierarchical cluster analysis on a cross-sectional sample of adults with SCD who are enrolled in the Globin Research Network Data and Discovery (GRNDaD) registry. Five hundred thirty-two participants (61% females and 64% with chronic pain) were included in the analysis. Six distinct subgroups were identified, 3 with chronic pain (Clusters 1–3) and 3 without chronic pain (Clusters 4–6). Despite differences in biological markers of disease severity such as genotype, hemoglobin and fetal hemoglobin percentage, chronic pain subgroups had comparable odds of reporting worse pain impact, suggesting that chronic pain has a disproportionate influence on SCD pain when compared to other factors. Longitudinal studies are needed to further validate these findings and to determine how these pain subgroups may change. Overall, our findings indicate that understanding and preventing chronic pain in SCD must be a top priority to improve the quality of life of those living with SCD.

## Introduction

Sickle cell disease (SCD) is the most common inherited hemoglobinopathy globally. Pain is the hallmark of SCD, and is associated with high healthcare utilization and premature mortality [1]. Acute, episodic severe pain evolves into chronic/persistent pain in 50%-60% of individuals by late adolescence to early adulthood [2,3]. Chronic SCD pain contributes to poor health-related quality of life in SCD [4,5]. However, despite the significant impact of SCD pain on public health and individual well-being, effective pain therapies are few, leading to disparities in pain outcomes.

Variability in pain symptoms among individuals with SCD has contributed to poor pain outcomes. This variability, present both across different individuals and within the same individual over time, includes variations in pain frequency and distribution, and is also noted among SCD subgroups with chronic/persistent pain [5–8], presenting considerable challenges in accurately phenotyping SCD pain. Consequently, this has limited our understanding of the etiology of chronic SCD pain, and has hindered the development of personalized treatment strategies. Gaining a better understanding of pain variability in SCD can inform more effective personalized approaches to managing chronic SCD pain. Similar approaches have been valuable in other chronic pain disorders, suggesting potential strategies for SCD [9–11].

Prior studies have used cluster analysis to identify patterns in pain symptoms and treatment response in adults suffering from various chronic pain disorders such as fibromyalgia, chronic low back pain, and temporomandibular disorder [9–16]. However, in SCD, attempts to identify homogenous pain subgroups using cluster analysis or similar machine learning methods have been limited [6,7,17]. Prior studies have shown that higher pain intensity and frequency are associated with worse psychosocial symptoms and lower quality of life. However, these studies included relatively small to moderate sample sizes, ranging from 62 to 291 participants, and only one study included participants from multiple centers. Moreover, only one of the three studies specifically examined individuals with chronic SCD pain, and none of the three studies compared the relationships between pain impact and both psychosocial and biological markers of disease severity.

Given the small number of studies, our understanding of interindividual variability in SCD pain remains limited. To address this gap, the primary objective of this current study was to identify distinct pain subgroups – both with and without chronic pain – by conducting a cluster analysis of a heterogeneous sample of adults with SCD enrolled in the Globin Research Network for Data and Discovery (GRNDaD) registry. We drew on findings from our previous work, which examined biopsychosocial variables associated with pain severity and pain impact among individuals with SCD, to select classification variables for the cluster analysis [3]. In that earlier work, we identified significant associations between psychosocial factors and both pain severity and pain impact – an SCD-specific measure of pain interference. For the current study, we hypothesized that while chronic pain would correlate with pain impact, differences in pain impact would emerge based on sex and biopsychosocial variables (e.g., total hemoglobin and emotional functioning), even within subgroups experiencing chronic pain. By identifying pain subgroups with shared demographic and biopsychosocial characteristics, we aim to deepen our understanding of pain variability among SCD participants with and without chronic pain, and to ultimately inform the development of more personalized and effective treatment strategies for chronic SCD pain.

## Materials and methods

### Study design and participants

**Fig 1:**
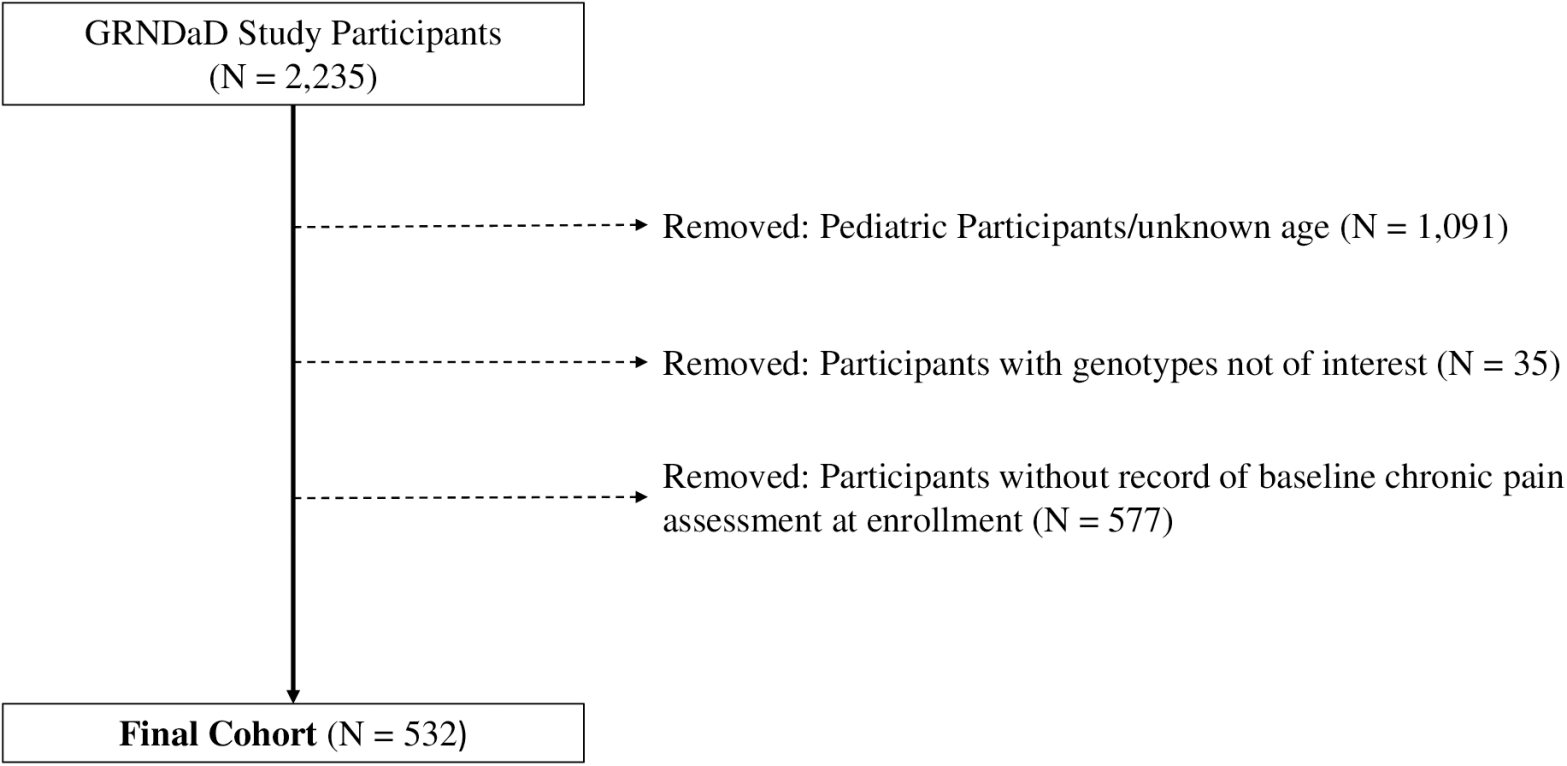
Flow diagram for inclusion of participants in analysis sample.

This study is a cross-sectional analysis of data from the multi-institutional GRNDaD registry (https://grndad.org) as of April 2022. The registry enrolls and longitudinally follows participants with laboratory-confirmed diagnosis of SCD. Detailed eligibility criteria for participants in the GRNDaD registry have been previously published. Our analysis was limited to adults 18 years or older with the most common SCD genotypes – HbSS, HbSC, and HbS-beta thalassemia – who are enrolled in GRNDaD and whose baseline assessment include a response to the presence or absence of chronic pain. Fig 1 illustrates cohort selection for this analysis. Five hundred thirty-two adults from 11 sickle cell centers were included in this analysis. When these participants were compared to 612 adults who were not included in the study, no significant differences were found in their sociodemographic or clinical characteristics, except for exclusion of rare genotypes, as shown in S1 Table in S1 File.

### Procedures

GRNDaD operates under a single institutional review board at Johns Hopkins University School of Medicine. Study personnel at each participating institution are responsible for obtaining written consent and enrolling participants. Enrollment for GRNDAD began January 2015 and remains ongoing. Data are securely stored in the Research Electronic Data Capture database (REDCap). Upon enrollment, participants complete validated self-reported questionnaires to assess pain, disease complications, and other patient-reported outcomes.

### Measures

#### Sociodemographic measures.

Age, sex, education level, household income, and race were self-reported upon enrollment.

#### Disease-specific measures.

Laboratory values, including hemoglobin and fetal hemoglobin percentage (HbF%), were obtained from GRNDaD during the same calendar year as participant questionnaires were completed. In most cases, these lab values were collected within one month of completing questionnaires.

***Pain-specific Measures.*** The primary outcome of interest is the Adult Sickle Cell Quality of Life Measurement Information System (ASCQ-Me) Pain Impact score [18], a 5-item scale that assesses pain interference over a 7-day recall period. This scale correlates with the Patient-Reported Outcomes Measurement Information System (PROMIS) Pain Interference and Pain Behavioral questionnaires, and is considered a disease-specific measure of pain interference [19]. Raw scores were converted to T-scores using conversation tables in the ASCQ-Me User’s Manual [20]. The score range is 0–100, with higher T-scores indicating better health or less pain impact.

A baseline assessment of chronic pain is included with enrollment in GRNDaD. Specifically, participants are asked if they experienced pain on most days during the preceding 6 months. This approach is consistent with the published consensus definition of chronic SCD pain [21].

The frequency of SCD pain episodes was determined using item 1 from the ASCQ-Me Pain Episodes Frequency and Severity Measure subscale.

#### Psychosocial measures.

Two psychosocial constructs, social and emotional functioning, were assessed using the ASCQ-Me Social Functioning Impact Measure and Emotional Impact Measure, both 5-item scales. The social functioning impact measure evaluates participation in social activities and roles, while the emotional impact measure assesses psychological distress, and correlates with PROMIS depression and anxiety scales. Raw scores were converted to T-scores (range 0–100), with higher scores indicating better function in the specific construct.

### Data analyses

In this study, we investigated pain variability between different individuals with SCD by first identifying and characterizing subgroups of GRNDaD participants using agglomerative hierarchical cluster analysis. We then examined the relationship between each identified cluster or subgroup and pain impact. Data were organized, cleaned, and analyzed using R statistical program (R Foundation for Statistical Computing, Vienna, Austria) version 4.3.2.

Our cluster analysis included 7 classification variables selected *a priori* based on evidence from the literature and our prior published analysis:[3] genotype (SS/Sβ^0^ or Variant [SC/Sβ^+^]), sex (male or female), baseline chronic pain (yes or no), age, maximum social and emotional functioning scores, and number of pain episodes (all from the same year as the worst pain impact score for individuals with repeated measures). Chronic pain was included as a classification variables because the clinical evidence indicated that variability in pain and pain-related outcomes is present even among SCD participants with chronic pain [5–7,17]. The rate of missingness was low for classification variables, and no missingness was found for genotype, age, sex, and baseline chronic pain. The remaining variables had a missingness of 10 percent or less, i.e., social functioning score (9.8%), emotional functioning score (10%), and number of pain episodes (9.8%).

We selected agglomerative hierarchical clustering due to its ability to provide an interpretable, tree-like structure that visualizes how individuals with similar pain characteristics are grouped at different levels of similarity. This method is particularly well-suited for our study because it does not require a pre-specified number of clusters, allowing for a data-driven determination of subgroup structure using the elbow plot and Gap statistic. Additionally, hierarchical clustering accommodates mixed data types (both continuous and categorical variables) when paired with Gower’s method, making it an appropriate choice given our classification variables. Ward’s minimum variance method was used as the linkage criterion to minimize within-cluster variance, ensuring compact and well-separated clusters.

We evaluated both an elbow plot and Gap statistic plot (S1 and S2 Figures in S1 File) to determine the appropriate number of clusters. Based on insight from both plots and an evaluation of the sample size of each cluster, we identified 6 distinct clusters.

Additional baseline and clinical variables of interest with missing rates ranging from 6%-33.8% were imputed after our cluster analysis, using the R package “mice” and our cluster variables (age, sex, genotype, chronic pain on enrollment, number of pain episodes, social, emotional and pain impact scores, and cluster number). We generated 5 imputed datasets, and pooled the results using Robin’s rules.

All baseline clinical and sociodemographic characteristics were summarized as median with interquartile range (IQR) and/or mean with standard deviation (SD) for continuous variables and as frequency with percentage for categorical variables. Differences between the 6 clusters were examined using Kruskal-Wallis rank sum tests and either Fisher Exact tests or Chi-Square tests for independence. Additional post-hoc comparisons were conducted for variables found to be significantly different between clusters. Finally, we conducted ordinal logistic regression to investigate the association between identified clusters and pain impact. We validated the model by assessing the proportional odds assumption. The reported results are odds ratios with 95% confidence intervals. To account for multiple comparisons, we adjusted the p-values using the Holm correction.

## Results

### Sample characteristics

The median age of the participants was 32 years (interquartile range [IQR] of 25–41 years), and 61% were female. Most participants (n = 370, 69.5%) had sickle cell anemia ([SCA], hemoglobin genotype HbSS or Sβ^0^), while approximately 31% had a variant genotype (hemoglobin SC or Sβ^+^). Chronic pain was reported by about 64% of the participants, and the median pain impact for the sample was 49.9 (IQR 44.4–58.0). Table 1 presents the baseline clinical and demographic characteristics of the sample.

**Table 1:** Demographic and clinical characteristics of participant sample (N, %) or median [IQR].

Characteristic	All Participants N = 532	Females N = 324 (60.9%)	Males N = 208 (39.1%)	P-value
Age				
Median [IQR]	32.00 [25.00, 41.00]	33.0 [26.00, 43.00]	30.00 [24.00, 40.00]	0.016
Mean(±SD)	34.52 (±12.47)	35.48(±12.64)	33.03 (±12.07)	0.027
Range	(18, 79)	(18, 79)	(18, 74)	
18-30	279 (45.6)	132 (40.7)	107 (51.4)	0.196
31-40	166 (27.1)	99 (30.6)	53 (25.5)	
41-50	100 (16.3)	45 (13.9)	24 (11.5)	
51-60	41 (6.7)	34 (10.5)	18 (8.7)	
61+	26 (4.2)	14 (4.3)	6 (2.9)	
Genotype				0.176
HbSS	347 (65.2%)	210 (64.8)	137 (65.9)	
HbSC	123 (23.1%)	76 (23.5)	47 (22.6)	
HbSβ^ + ^-thalassemia	39 (7.3%)	28 (8.6)	11 (5.3)	
HbSβ^0^-thalassemia	23 (4.3%)	10 (3.1)	13 (6.2)	
Hemoglobin g/dL	9.6 [8.20, 10.92]	9.00 [7.90, 10.60]	10.20 [8.80, 11.80]	<0.001
Fetal hemoglobin %	4.45 [1.67, 10.80]	4.75 [1.90, 11.07]	3.90 [1.50, 10.02]	0.168
Hydroxyurea	362 (68.2)	212 (65.6)	150 (72.12)	0.142
Chronic Pain (on enrollment)*	342 (64.3)	211 (65.1)	131 (63.0)	0.681
Pain Episodes				0.244
None	60 (12.5)	35 (11.9)	25 (13.4)	
1	53 (11.0)	34 (11.6)	19 (10.2)	
2	75 (15.6)	41 (13.9)	34 (18.3)	
3	92 (19.2)	65 (22.1)	27 (14.5)	
4 or more	200 (41.7)	119 (40.5)	81 (43.5)	
Emotional Functioning^†^	51.50 [46.20, 57.30]	51.50 [44.90, 57.30]	53.30 [47.40, 60.50]	0.016
Social Functioning^†^	52.20 [45.60, 59.80]	50.50 [43.90, 57.70]	54.00 [47.20, 59.80]	0.008
Pain Impact^§^	49.90 [44.40, 58.0]	49.90 [44.40, 58.00]	49.90 [44.40, 60.90]	0.470
Highest level of education completed				0.148
Grades 1–11	24 (5.8)	11 (4.3)	13 (8.2)	
High School/GED	168 (40.8)	97 (38.3)	71 (44.7)	
Some College/Associates	112 (27.2)	73 (28.9)	39 (24.5)	
College degree and above	108 (26.2)	72 (28.5)	36 (22.6)	
Income (enrollment year)				0.331
Less than $12,060	214 (44.4)	126 (42.9)	88 (46.8)	
$12,060 to $16,240	129 (26.8)	76 (25.9)	53 (28.2)	
More than $28,780	139 (28.8)	92 (31.3)	47 (25.0)	
Admissions (enrollment year)				0.352
0	221 (44.4)	127 (42.1)	94 (48.0)	
1-3	189 (38.0)	117 (38.7)	72 (36.7)	
4-6	54 (10.8)	35 (11.6)	19 (9.7)	
7-12	27 (5.4)	20 (6.6)	7 (3.6)	
13+	7 (1.4)	3 (1.0)	4 (2.0)	
Baseline ED visits				0.271
0	221 (44.4)	129 (42.9)	93 (47.9)	
1-3	189 (38.0)	116 (38.5)	71 (36.6)	
4-6	54 (10.8)	33 (11.0)	11 (5.7)	
7-12	27 (5.4)	11 (3.7)	9 (4.6)	
13+	7 (1.4)	12 (4.0)	10 (5.2)	

Abbreviations: IQR, interquartile range; GED, General Educational Development

*Self-report measure

† T-score. Higher value indicates better health in domain

§ T-score. Higher value indicates less pain impact (better health)

Some notable differences were observed between females (n = 324) and males (n = 208) in the cohort. Males were younger, with a median age of 30 years (IQR 24–40), compared to females with a median age of 33 years (IQR 26–43), p = 0.016. Females also had lower median hemoglobin levels (7.9 g/dL) than males (10.2 g/dL), p < 0.001, and slightly lower social and emotional functioning scores than males, i.e., 51.5 vs 53.3, p = 0.016; and 50.5 vs 54, p = 0.008, respectively. Differences in chronic pain prevalence and other sociodemographic and clinical variables were not significant (**Table 1**). In addition to no significant difference in median pain impact scores among males (49.90 [44.40, 60.90]) and females (49.90 [44.40, 58.00]) (p = 0.470), we also noted no significant effect of sex on pain impact (OR = 0.91 [95% CI: 0.66, 1.27], p = 0.589). Further, in our model with sex, chronic pain and an interaction between the two, both sex (OR = 0.68 [95% CI: 0.37, 1.24], p = 0.207) and the interaction were insignificant (OR = 1.54 [95% CI: 0.74, 3.21], p = 0.248), though chronic pain was significant (OR = 11.11 [95% CI: 6.73, 18.33], p < 0.001).

### Description and characteristics of clusters

Six distinct clusters, or pain subgroups, were identified – 3 with chronic pain (Clusters 1–3) and 3 without chronic pain (Clusters 4–6). Each classification variable differed significantly across the 6 clusters (**Table 2**).

**Table 2:** Characterization of the six pain subgroups (“clusters”) with sickle cell disease N(%) or median [IQR].

	Cluster 1	Cluster 2	Cluster 3	Cluster 4	Cluster 5	Cluster 6	P-value
*N*	*134*	*116*	*92*	*58*	*46*	*86*	
**Sickle cell subtype**							<0.001
SCA (SS/Sβ^0^)	134 (100)	0 (0)	92 (100)	58 (100)	0 (0)	86 (100)	
Variant (SC/Sβ^+^)	0 (0)	116 (100)	0 (0)	0 (0)	46 (100)	0 (0)	
**Sex**							<0.001
Female	134 (100)	77 (66.4)	0 (0)	0 (0)	27 (58.7)	86 (100)	
Male	0 (0)	39 (33.6)	92 (100)	58 (100)	19 (41.3)	0 (0)	
**Age**	32 [27.00, 39.00]	35.50 [26.75, 47.00]	30.0 [25.00, 37.25]	24.0 [21.25, 34.75]	38.0 [27.25, 51.75]	33.0 [23.0, 40.75]	<0.001
**Pain Episodes**							<0.001
0	5 (4.2)	5 (4.6)	7 (8.4)	9 (18.4)	14 (35.0)	20 (25.0)	
1	7 (5.8)	9 (8.3)	6 (7.2)	9 (18.4)	7 (17.5)	15 (18.8)	
2	15 (12.5)	18 (16.7)	13 (15.7)	11 (22.4)	6 (15.0)	12 (15.0)	
3	33 (27.5)	23 (21.3)	7 (8.4)	10 (20.4)	4 (10.0)	15 (18.8)	
4+	60 (50.0)	53 (49.1)	50 (60.2)	10 (20.4)	9 (22.5)	18 (22.5)	
**Emotional Functioning**	48.70 [44.60, 55.20]	50.10 [44.90, 53.30]	50.10 [45.55, 55.20]	60.50 [55.20, 65.60]	57.30 [48.38, 61.77]	56.25 [50.10, 60.50]	<0.001
**Social Functioning**	48.80 [43.90, 52.65]	47.20 [42.10, 52.20]	50.50 [45.60, 57.70]	62.10 [57.70, 69.80]	59.80 [50.50, 64.90]	59.80 [53.55, 69.80]	<0.001
**Chronic Pain**	134 (100.0)	116 (100.0)	92 (100)	0 (0)	0 (0)	0 (0)	<0.001

**Cluster 1: Female only, Chronic Pain, SCA** – One hundred and thirty-four (25%) of the participants were included in Cluster 1. This cluster consisted solely of female participants with SCA, all reporting chronic pain. Half experienced 4 or more pain episodes in a 12-month period, and their social/emotional functioning scores were below average.

**Cluster 2: 66% Female, Chronic Pain, Variant Genotype** – One hundred sixteen participants (22%) comprised Cluster 2, which included only individuals with HbSC or HbSβ^+^ (variant genotypes) and chronic pain. Of these, 66% were female. This group was marked by frequent painful episodes and low social/emotional functioning scores.

**Cluster 3: Male only, Chronic Pain, SCA** – Ninety-two participants (17%) were grouped into Cluster 3. All were males with SCA and chronic pain. Social/emotional functioning scores were low.

**Cluster 4: Male only, No Chronic Pain, SCA** – Fifty-eight participants (11%) comprised Cluster 4, which was made up entirely of males with SCA and *no* chronic pain. Pain episodes were infrequent and social/emotional functioning scores were high.

**Cluster 5: 59% Female, No Chronic Pain, Variant Genotype** – Forty-six participants (8.6%) comprised Cluster 5. Approximately 59% were females. Chronic pain was absent in this cluster. Social/emotional functioning scores were high.

**Cluster 6: Female only, No Chronic Pain, SCA** – Eighty-six participants (16%) were included in Cluster 6. This cluster consisted solely of female participants with SCA and no chronic pain. Pain episodes were infrequent, and social/emotional functioning scores were high.

### Comparison between pain clusters

#### Pain impact.

**Fig 2:**
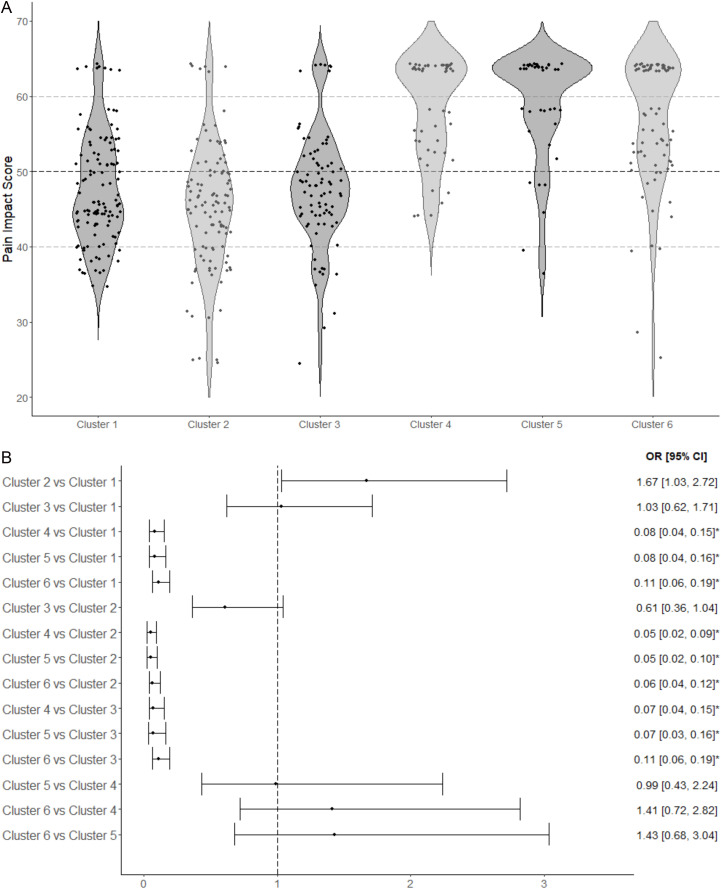
Panels A and B depict the relationship between clusters, or subgroups, and pain impact. Pain impact assessed by ASCQ-Me Pain Impact Short Form (5-item questionnaire). Raw scores converted to T-scores. Higher T-scores imply better health or less pain impact. ***A.*** Plot depicting the distribution of pain impact T-scores for each cluster. ***B.*** Ordinal logistic regression modeling comparing the association of each cluster with pain impact.

Distribution of median pain impact scores across the 6 clusters is depicted in **Fig 2A**. Most of the participants in the chronic pain subgroups (Clusters 1–3) had median pain impact scores below 50 (i.e., greater pain impact). Cluster 2 had the largest percentage of participants (76.9%) with greater pain impact, that is pain impact scores at or below 50. The subgroups without chronic pain (Clusters 4–6) had higher pain impact scores, indicating better health or less pain impact, and only a small subset of participants had scores at or below 50.

We assessed the relationship between the identified clusters and pain impact. Among Clusters 4–6 (no chronic pain), Cluster 6 appeared to have greater odds of higher pain impact compared to 4 or 5; however, the differences between these clusters were not statistically significant. When corrected for multiple comparisons, no statistically significant differences in pain impact between the 3 chronic pain subgroups were found (**Fig 2B**). Cluster 2 appeared to have increased odds of having more severe pain impact (i.e., lower scores) compared to Cluster 1 (OR = 1.67 [95% CI: 1.03, 2.72]).

However, significant differences in pain impact were noted between each subgroup with no chronic pain and each subgroup with chronic pain. For instance, compared to Cluster 1 (female only, chronic pain, SCA), Cluster 6 (female only, no chronic pain, SCA) had significantly lower odds of experiencing more severe pain impact, with an odds ratio of 0.11 (95% CI: 0.06, 0.19, p < 0.001). This relationship was consistent in all comparisons between clusters without chronic pain vs clusters with chronic pain, with the clusters without chronic pain significantly less likely to report higher pain impact.

#### Additional variables.

We conducted detailed post-hoc comparisons of additional variables of interest (hydroxyurea use at the time of enrollment, admissions and emergency department visits in the 12 months preceding enrollment, education, income, total hemoglobin, and HbF%) to assess differences between the 6 clusters (S2 and S3 Tables in S1 File). Hydroxyurea use differed significantly between clusters with SCA (Clusters 1, 3, 4, and 6) and clusters with variant genotypes (Clusters 2 and 5), with approximately 80%-86% of participants with SCA on hydroxyurea. Similar patterns emerged when comparing hemoglobin levels, as median hemoglobin was significantly higher among participants in Clusters 2 and 5 (variant genotypes) vs participants in Clusters 1, 3, 4, or 6. No significant difference in hemoglobin levels was noted between Clusters 2 and 5. When examining HbF% levels, significant differences were noted only between Cluster 2 and Clusters 1, 3, 4, or 6. Compared to these 4 clusters, Cluster 2 had a significantly lower median HbF%. Percentage fetal hemoglobin, however, did not differ significantly between Clusters 2 and 5 (variant genotypes). No significant differences in HbF% were noted between Cluster 5 (variant genotype and no chronic pain) and Clusters 1, 3, 4, or 6.

Baseline admissions were significantly different between Clusters 1 and 6. Additionally, income varied significantly between Clusters 1 and 2, 1 and 5, 2 and 3, and 2 and 4 as well as 2 and 6, and 4 and 5. After adjusting for multiple comparisons, no significant differences in ED visits or education were observed between any 2 clusters.

## Discussion

Here, we report the results of an analysis aimed at understanding variability in SCD pain experiences, using data from a multicenter SCD registry. Six distinct subgroups – 3 with chronic pain and 3 without chronic pain – were identified, highlighting the complexity and heterogeneity of pain experiences within the SCD population. As expected, subgroups with high baseline chronic pain (Clusters 1–3) differed significantly in pain impact compared to those without chronic pain (Clusters 4–6). While this finding aligns with our hypothesis and existing literature, the key significance of our study lies in its novel contribution to the SCD pain literature. Specifically, our study is one of the few to highlight the substantial burden of chronic pain in SCD through a detailed cluster or subgroup analysis, revealing that the presence of chronic pain predominates over other known associations with pain impact. These findings emphasize the critical importance of chronic pain prevention in SCD, particularly in the context of recent advances in disease-modifying and curative therapies. The results suggest that a greater focus on treating chronic pain may have a disproportionate impact on pain outcomes in SCD, and chronic pain prevention should be integrated as a central element in the overall care strategy for individuals with SCD.

Specifically, our analysis revealed that biological variables such as hemoglobin, SCD genotype, and HbF%, poorly accounts for the differences noted in high vs low pain impact subgroups, which extends the findings from our prior work [3]. For example, despite significant differences in biological variables (i.e., genotype, hemoglobin, and HbF%), Clusters 1 and 2 exhibited similar pain impact scores. Moreover, Clusters 2 and 5 shared similar biological profiles (i.e., same genotype and no significant differences in hemoglobin or HbF%), but differed significantly in pain profiles, primarily due to the presence or absence of chronic pain. Altogether, these findings suggest that chronic pain exerts a significant influence on pain outcomes in SCD, independent of traditional biological markers of disease severity – a relationship that is not well established in the context of SCD.

To the best of our knowledge, this is the largest study yet to use cluster analysis or machine learning methods to examine variability in pain experiences among individuals living with SCD. This study advances prior pain variability research in SCD [6,7,17] by using a larger multicenter cohort and emphasizes the pivotal role of chronic pain in driving pain outcomes, rather than relying solely on biological markers of disease severity. Additionally, our study addresses a gap in the literature by examining sex-related differences in pain impact within the context of chronic pain. Some prior studies have reported higher pain frequency and severity among females with SCD [8,22,23]. An analysis of 2,124 participants enrolled in the Sickle Cell Disease Implementation Consortium (SCDIC) registry, found that females with SCD were more likely to report higher pain frequency and pain severity [23]. A smaller, but longitudinal, study reported no sex-related differences in pain frequency or intensity [8]. Other studies have reported a greater prevalence of anxiety and depression among females with SCD compared to males, conditions often associated with worse pain [8,24,25]. However, these prior studies did not consider the presence or absence of chronic pain in their assessment of sex-related differences. Contrary to these studies, our analysis did not find significant differences in pain impact between male and female subgroups with similar genotypes and chronic pain prevalence. For example, Cluster 1, made up of females with SCA and chronic pain, and Cluster 3, comprised of males with SCA and chronic pain, had comparable pain impact scores. These observations were also present between Cluster 4 (females with SCA and no chronic pain) and Cluster 6 (males with SCA and no chronic pain). The lack of a significant effect of sex on pain impact was further confirmed by our regression models, which indicated that sex is not moderated by chronic pain. Our findings suggest that sex alone does not predict pain impact in our cohort of adults living with SCD, further emphasizing the need for personalized approaches to pain management that consider the interplay of multiple biopsychosocial factors.

Interestingly, we noted significant differences only in hospital admissions between Clusters 1 and 6, but no differences in ED visits between other clusters. However, this is consistent with findings from the Sil et al study [6], which examined variability in chronic pain in 62 children living with SCD. Differences in sociodemographic characteristics, specifically income and education, were variable in comparisons between clusters. A general pattern was observed in which participants in the clusters with a variant genotype had significantly higher income when compared to participants in clusters with SCA. This difference may be attributed to the difference in life expectancy and overall reduced risk for end-organ complications in individuals with a variant genotype when compared to those with SCA.

The key contribution of our study is the demonstration that chronic pain has an oversized influence on pain outcomes in SCD. Therefore, the prevention of chronic pain must accompany efforts at disease modification in SCD. Recently developed medications for SCD – L-glutamine, crizanlizumab, and voxelotor – have not consistently shown reductions in pain intensity or impact [26–30]. Over the past decade, transformative therapies for SCD, such as gene therapy and hematopoietic stem cell transplantation (HSCT), have been developed to eliminate or mitigate the effects of sickle hemoglobin by directly targeting the pathophysiology of SCD. The outcomes have been dramatic, with marked reductions in acute pain episodes and healthcare utilization, and improvements in quality of life, alongside improvements in biological parameters such as hemoglobin, hemolysis, and cerebral blood flow, a risk factor for strokes [31–34]. However, chronic pain post curative therapy persists in many individuals, with up to 40% and 18% still requiring opioids within the first and second years after treatment, respectively [32,35]. A few individuals continue to have episodic “vaso-occlusive” pain following these therapies. The impact of these therapies on chronic SCD pain, which is likely mediated by central mechanisms [36,37], remains to be fully understood. In addition to the unclear impact of curative therapy on chronic SCD pain, concerns such as donor availability, cost of therapy, and unknown long-term prognosis after gene therapy remain [26,38–41]. These limitations, in the context of our findings, do not diminish the tremendous impact of these advanced therapies for the individuals who receive them, but rather highlight the need for a better understanding and management of chronic pain in SCD. Our findings in the current study, combined with emerging research on outcomes following curative therapy, suggest that once chronic pain has developed, disease modification alone may not suffice to reduce pain severity and interference in many individuals living with SCD. Long-term pain outcomes for individuals who receive these therapies warrant further investigation, ideally through the systematic collection and analysis of longitudinal data from resources such as the GRNDaD registry. A deeper understanding of chronic pain and pain variability in SCD could facilitate the development of new and effective preventive pain interventions, and assist in identifying individuals who would benefit most from specific therapeutic interventions such as gene therapy.

While our study highlights the significant role of chronic pain in pain experiences among individuals with SCD, we were limited in the range of psychosocial variables we could examine. In a prior pain subgrouping study, Sil et al focused exclusively on pediatric patients with chronic SCD pain, and reported significant differences in psychosocial characteristics among the 3 pain clusters identified in their study. Psychosocial factors such as self-efficacy and pain coping strategies, are known to influence pain outcomes in other painful disorders [42]. While these factors are also believed to influence pain in SCD [4,43,44], their relative importance compared to other sociodemographic and biopsychological variables must be further investigated. Understanding protective factors by studying pain subgroups, such as Clusters 4 and 5, is critical to the design and implementation of preventive pain interventions for the SCD population.

### Strengths and limitations

The interpretation of findings from this study should consider several limitations. The study was a secondary analysis of registry data, and thus, limits our ability to establish causality or predict pain outcomes. The reliance on self-report measures may introduce recall and nonresponse biases. While the classification and outcome variables used in analysis were informed by our prior research, they were limited to variables collected in GRNDaD, which may not fully capture the complexity of the pain experience in SCD. Additional subgroups or other clinically relevant distinctions between subgroups may have emerged if additional biopsychosocial measures known to influence pain outcomes had been included in the analysis. For example, incorporating specific pain measures such as number of pain locations or pain quality, may have revealed additional distinctions between the subgroups and potentially identified new subgroups. Furthermore, understanding the impact of early life experiences, such as childhood hospitalizations or behavioral health history could provide deeper insights into the development and persistence of chronic SCD pain [45]. Additional limitations include variations across SCD centers, especially regarding funding, resources, and the availability of non-pharmacologic pain treatments (e.g., acupuncture and cognitive behavioral therapy).

### Conclusions & future directions

Our results here strongly indicate that the underlying cause of chronic pain must be addressed to significantly lower the burden of pain in SCD even as advancements in curative therapy continue. Preventing chronic pain must be a top priority aimed to improve quality of life for people living with SCD. Future research should prioritize our understanding of the underlying cause of chronic pain, and risk and protective factors for developing chronic pain in SCD. Additionally, further research is required to understand long-term pain outcomes in SCD in order to inform the development and refinement of preventive and symptomatic interventions for chronic SCD pain, and to identify individuals who would most likely benefit from emerging therapies.

## Supporting information

S1 File S1 FIGURE – Dendrogram from agglomerative hierarchical clustering using Ward’s method, illustrating the hierarchical structure of pain subgroups in individuals with sickle cell disease. Colored boxes indicate the identified clusters at the selected cutoff level. S2 FIGURE – Assessment of agglomerative clustering solutions using (A) the within-cluster sum of squares (elbow method) and (B) the gap statistics. Dashed lines indicate the suggested optimal number of clusters based on each method. S1 TABLE – Demographic and clinical characteristics of the participant sample, including comparisons between included and excluded participants. S2 TABLE – Clinical and sociodemographic characteristics of the identified pain subgroups. S3 TABLE – Post-hoc comparisons of clinical and sociodemographic characteristics between pain subgroups.
(PDF)
