# Supplementary material for: Identifying distinct subgroups with severe pain in sickle cell disease: A cluster analysis of the GRNDaD multi-center registry
Source: PLoS One. 2025 May 15;20(5):e0320889. doi: 10.1371/journal.pone.0320889 (PMC12080796; doi:10.1371/journal.pone.0320889)
Supplement: S1 File — Colored boxes indicate the identified clusters at the selected cutoff level. S2 FIGURE – Assessment of agglomerative clustering solutions using (A) the within-cluster sum of squares (elbow method) and (B) the gap statistics. Dashed lines indicate the suggested optimal number of clusters based on each method. S1 TABLE – Demographic and clinical characteristics of the participant sample, including comparisons between included and excluded participants. S2 TABLE – Clinical and sociodemographic characteristics of the identified pain subgroups. S3 TABLE – Post-hoc comparisons of clinical and sociodemographic characteristics between pain subgroups. (PDF) [file pone.0320889.s001.pdf]

Agglomerative clustering, Ward D

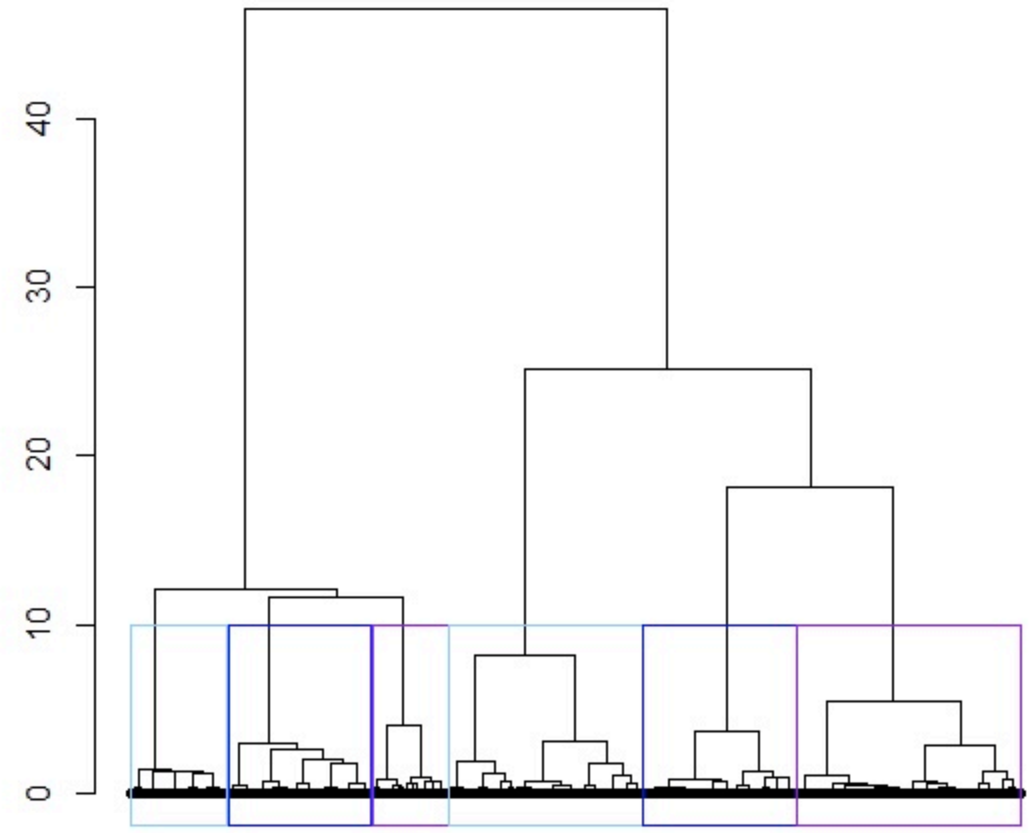

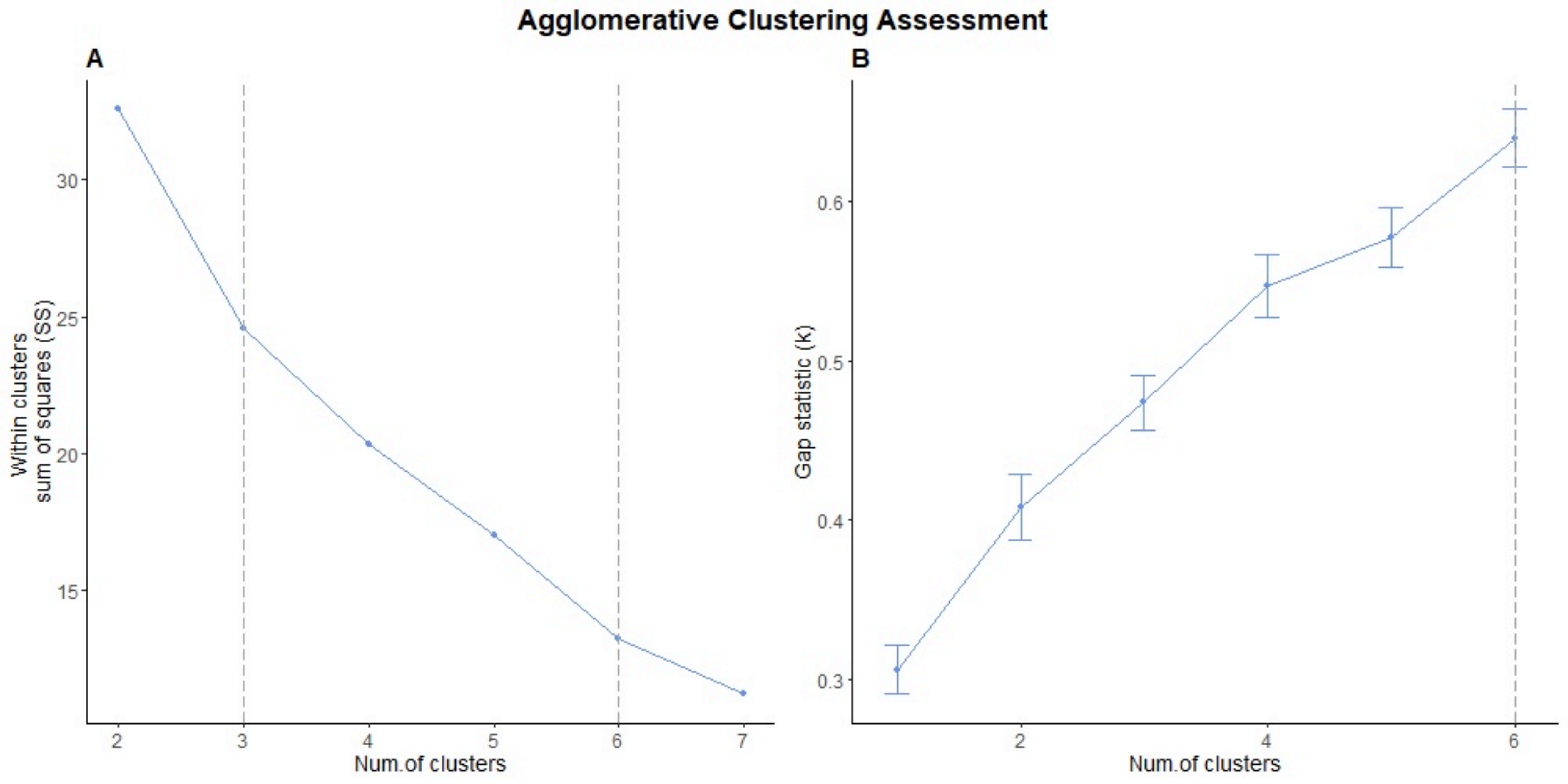
Agglomerative Clustering Assessment
A
B
Within clusters
sum of squares (SS)
30
25
20
15
2
3
4
5
6
7
Num.of clusters
Gap statistic (k)
0.6
0.5
0.4
0.3
2
4
6
Num.of clusters

TABLE S1. Demographic and clinical characteristics of participant sample (N, %) or median [IQR]

Characteristic	Included Participants N = 532	Excluded Participants N = 612	P-value
Age			
Median [IQR]	32.00 [25.00, 41.00]	32.00 [24.75, 41.00]	0.910
Mean±SD	34.52 ±12.47	34.40 (±11.95)	0.876
Range	(18, 79)	(18, 76)	
18-30	279 (45.6)	239 (44.9)	0.206
31-40	166 (27.1)	152 (28.6)	
41-50	100 (16.3)	69 (13.0)	
51-60	41 (6.7)	52 (9.8)	
61+	26 (4.2)	20 (3.8)	
Missing	*0*	*0*	
Genotype			<0.001
SCA (SS/Sβ^0)	370 (69.5)	392 (64.9)	
Variant (SC/Sβ+)	162 (30.5)	185 (30.6)	
Other	0	27 (4.5)	
Missing	*0*	*8*	
Hemoglobin g/dL	9.6 [8.20, 10.92]	9.40 [8.30, 10.35]	0.543
Missing	*82*	*541*	
Fetal hemoglobin %	4.45 [1.67, 10.80]	6.35 [1.87, 15.65]	0.327
Missing	*180*	*564*	
Hydroxyurea	362 (68.2)	403 (67.2)	0.766
Missing	*1*	*12*	
Chronic Pain (on enrollment)*	342 (64.3)	13 (65.0)	>0.999
Missing	*0*	*592*	
Pain Episodes			0.360
None	60 (12.5)	10 (12.7)	
1	53 (11.0)	5 (6.3)	
2	75 (15.6)	19 (24.1)	
3	92 (19.2)	15 (19.0)	
4 or more	200 (41.7)	30 (38.0)	
Missing	*52*	*533*	
Emotional Functioning[†]	51.50 [46.20, 57.30]	53.30 [46.80, 57.30]	0.885
Missing	*53*	*533*	
Social Functioning[†]	52.20 [45.60, 59.80]	50.50 [47.20, 55.80]	
Missing	*52*	*535*	
Pain Impact[§]	49.90 [44.40, 58.0]	48.50 [44.0, 60.90]	
Missing	*51*	*533*	
Highest level of education completed			0.420
Grades 1 through 11	24 (5.8)	3 (9.4)	
High School/GED	168 (40.8)	15 (46.9)	
Some College/Associates	112 (27.2)	5 (15.6)	
College degree and above	108 (26.2)	9 (28.1)	

TABLE S1. Demographic and clinical characteristics of participant sample (N, %) or median [IQR]

Characteristic	Included Participants N = 532	Excluded Participants N = 612	P-value
Missing	*120*	*580*	
Income (enrollment year)			0.344
Less than $12,060	214 (44.4)	10 (40.0)	
$12,060 to $16,240	129 (26.8)	10 (40.0)	
More than $28,780	139 (28.8)	5 (20.0)	
Missing	*50*	*587*	
Admissions (enrollment year)			0.712
0	221 (44.4)	198 (43.0)	
1-3	189 (38.0)	173 (37.5)	
4-6	54 (10.8)	47 (10.2)	
7-12	27 (5.4)	35 (7.6)	
13+	7 (1.4)	8 (1.7)	
Missing	*34*	*116*	
Baseline ED visits			0.672
0	221 (44.4)	188 (40.3)	
1-3	189 (38.0)	186 (39.9)	
4-6	54 (10.8)	45 (9.7)	
7-12	27 (5.4)	24 (5.2)	
13+	7 (1.4)	23 (4.9)	
Missing	*37*	*111*	

Abbreviations: IQR, interquartile range; GED, General Educational Development
*Self-report measure
†T-score. Higher value indicates better health in domain
§T-score. Higher value indicates less pain impact (better health)

TABLE S2. Clinical and sociodemographic characteristics of clusters (N, %)

	Cluster 1	Cluster 2	Cluster 3	Cluster 4	Cluster 5	Cluster 6	P-value
N	*134*	*116*	*92*	*58*	*46*	*86*	
**Hydroxyurea** (%)	111 (83.5)	44 (37.9)	78 (84.8)	50 (86.2)	7 (15.2)	72 (83.7)	<0.001[1]
missing	*1*	*0*	*0*	*0*	*0*	*0*	
**Income** (%)							<0.001[1]
Less than $12,060	62 (51.7)	46 (42.6)	52 (59.8)	18 (36.7)	10 (23.8)	26 (34.2)	
$12,060 to $28,780	40 (33.3)	28 (25.9)	23 (26.4)	16 (32.7)	9 (21.4)	13 (17.1)	
More than $28,780	18 (15.0)	34 (31.5)	12 (13.8)	15 (30.6)	23 (54.8)	37 (48.7)	
missing	*14*	*8*	*5*	*9*	*4*	*10*	
**Highest Education** (%)							0.004[2]
Grades 1 through 11	2 (1.9)	3 (3.2)	10 (12.8)	3 (7.7)	3 (8.8)	3 (4.7)	
High School/GED	49 (47.1)	35 (37.6)	30 (38.5)	22 (56.4)	11 (32.4)	21 (32.8)	
Some College/Associates	32 (30.8)	30 (32.3)	24 (30.8)	5 (12.8)	7 (20.6)	14 (21.9)	
College degree and above	21 (20.2)	25 (26.9)	14 (17.9)	9 (23.1)	13 (38.2)	26 (40.6)	
missing	*30*	*23*	*14*	*19*	*12*	*22*	
**Baseline Admissions** (%)							0.008[2]
0	43 (33.1)	52 (50.0)	34 (38.2)	25 (46.3)	21 (51.2)	46 (57.5)	
1 to 3	48 (36.9)	39 (37.5)	34 (38.2)	23 (42.6)	17 (41.5)	28 (35.0)	
4 to 6	21 (16.2)	9 (8.7)	12 (13.5)	5 (9.3)	2 (4.9)	5 (6.2)	
7 to 12	16 (12.3)	3 (2.9)	5 (5.6)	1 (1.9)	1 (2.4)	1 (1.2)	
13+	2 (1.5)	1 (1.0)	4 (4.5)	0 (0.0)	0 (0.0)	0 (0.0)	
missing	*4*	*12*	*3*	*4*	*5*	*6*	
**Baseline ED Visits** (%)							0.016[2]
0	48 (36.9)	46 (44.7)	34 (39.5)	30 (54.5)	15 (35.7)	49 (62.0)	
1 to 3	51 (39.2)	39 (37.9)	32 (37.2)	22 (40.0)	23 (54.8)	20 (25.3)	
4 to 6	17 (13.1)	8 (7.8)	7 (8.1)	2 (3.6)	2 (4.8)	8 (10.1)	
7 to 12	5 (3.8)	5 (4.9)	6 (7.0)	1 (1.8)	2 (4.8)	1 (1.3)	
13+	9 (6.9)	5 (4.9)	7 (8.1)	0 (0.0)	0 (0.0)	1 (1.3)	
missing	*4*	*13*	*6*	*3*	*4*	*7*	
**Hemoglobin** (median [IQR])	8.33 [7.60, 9.63]	11.21 [10.38, 12.03]	9.40 [8.40, 10.40]	9.40 [8.20, 10.60]	11.10 [10.08, 12.15]	8.60 [7.77, 9.60]	<0.001[3]
missing	*19*	*16*	*12*	*13*	*8*	*14*	
**HBF** (median [IQR])	6.20 [2.73, 11.52]	1.60 [1.00, 3.70]	5.00 [1.95, 11.20]	5.35 [2.68, 12.45]	2.45 [1.47, 5.70]	8.90 [2.80, 17.42]	<0.001[3]
missing	*40*	*43*	*25*	*18*	*24*	*30*	

Abbreviations: HBF, percentage fetal hemoglobin
[1]Chi-square test, [2]Fisher exact test, [3]Kruskal-wallis rank sum test, [4]Independent samples t-test

Table S3: Post-hoc comparisons of clinical and sociodemographic characteristics of clusters

Cluster Comparison	1 vs 2	1 vs 3	1 vs 4	1 vs 5	1 vs 6	2 vs 3	2 vs 4	2 vs 5	2 vs 6	3 vs 4	3 vs 5	3 vs 6	4 vs 5	4 vs 6	5 vs 6	Status
**Genotype**[2]	<0.001	--	--	<0.001	--	<0.001	<0.001	--	<0.001	--	<0.001	--	<0.001	--	<0.001	Unadjusted
	0.004	--	--	0.004	--	0.004	0.004	--	0.004	--	0.004	--	0.004	--	0.004	Adjusted
**Sex**[2]	<0.001	<0.001	<0.001	<0.001	--	<0.001	<0.001	0.363	<0.001	--	<0.001	<0.001	<0.001	<0.001	<0.001	Unadjusted
	0.006	0.006	0.006	0.006	--	0.006	0.006	0.363	0.006	--	0.006	0.006	0.006	0.006	0.006	Adjusted
**Age**[3]	0.074	0.061	<0.001	0.067	0.224	0.002	<0.001	0.339	0.021	0.033	0.005	0.243	<0.001	0.007	0.024	Unadjusted
	0.365	0.365	0.006	0.365	0.671	0.029	<0.001	0.671	0.191	0.229	0.055	0.671	0.001	0.075	0.192	Adjusted
**Age Group**[2]	0.618	0.171	0.013	0.061	0.437	0.052	0.006	0.319	0.554	0.247	0.009	0.242	0.013	0.149	0.250	Unadjusted
	>0.999	>0.999	0.169	0.615	>0.999	0.577	0.090	>0.999	>0.999	>0.999	0.133	>0.999	0.169	>0.999	>0.999	Adjusted
**Pain Episodes**[2]	0.722	0.013	<0.001	<0.001	<0.001	0.113	0.002	<0.001	<0.001	<0.001	0.001	<0.001	0.345	0.807	0.696	Unadjusted
	>0.999	0.081	0.007	0.007	0.007	0.565	0.017	0.007	0.007	0.007	0.008	0.007	>0.999	>0.999	>0.999	Adjusted
**Emotional**[3]	0.250	0.290	<0.001	<0.001	<0.001	0.125	<0.001	<0.001	<0.001	<0.001	<0.001	<0.001	0.085	0.042	0.455	Unadjusted
	0.751	0.751	<0.001	<0.001	<0.001	0.499	<0.001	<0.001	<0.001	<0.001	0.002	<0.001	0.425	0.249	0.751	Adjusted
**Social**[3]	0.040	0.122	<0.001	<0.001	<0.001	0.003	<0.001	<0.001	<0.001	<0.001	<0.001	<0.001	0.026	0.021	0.402	Unadjusted
	0.119	0.244	<0.001	<0.001	<0.001	0.019	<0.001	<0.001	<0.001	<0.001	0.001	<0.001	0.107	0.107	0.402	Adjusted
**Baseline Chronic Pain**[2]	--	--	<0.001	<0.001	<0.001	--	<0.001	<0.001	<0.001	<0.001	<0.001	<0.001	--	--	--	Unadjusted
	--	--	0.004	0.004	0.004	--	0.004	0.004	0.004	0.004	0.004	0.004	--	--	--	Adjusted
**Hemoglobin**[3*]	<0.001	0.002	0.001	<0.001	0.150	<0.001	<0.001	0.311	<0.001	0.227	<0.001	0.195	<0.001	0.054	<0.001	Unadjusted
	<0.001	0.014	0.005	<0.001	0.599	<0.001	<0.001	0.599	<0.001	0.599	<0.001	0.599	0.001	0.270	<0.001	Adjusted
**HBF**[3*]	<0.001	0.085	0.188	0.039	0.073	<0.001	<0.001	0.167	<0.001	0.326	0.141	0.154	0.143	0.216	0.146	Unadjusted
	<0.001	0.768	>0.999	0.431	0.731	<0.001	0.003	>0.999	0.003	>0.999	>0.999	>0.999	>0.999	>0.999	>0.999	Adjusted
**Hydroxyurea**[1*]	<0.001	0.935	0.792	<0.001	>0.999	<0.001	<0.001	0.009	<0.001	0.997	<0.001	>0.999	<0.001	0.865	<0.001	Unadjusted
	<0.001	>0.999	>0.999	<0.001	>0.999	<0.001	<0.001	0.062	<0.001	>0.999	<0.001	>0.999	<0.001	>0.999	<0.001	Adjusted
**Income**[1*]	0.018	0.473	0.058	<0.001	<0.001	0.014	0.705	0.027	0.063	0.025	<0.001	<0.001	0.085	0.076	0.354	Unadjusted
	0.177	>0.999	0.403	<0.001	<0.001	0.153	>0.999	0.223	0.403	0.223	<0.001	<0.001	0.403	0.403	>0.999	Adjusted
**Admissions**[2*]	0.034	0.314	0.051	0.034	0.002	0.242	0.879	0.797	0.621	0.382	0.196	0.020	0.768	0.548	0.806	Unadjusted
	0.444	>0.999	0.557	0.444	0.027	>0.999	>0.999	>0.999	>0.999	>0.999	>0.999	0.281	>0.999	>0.999	>0.999	Adjusted
**Education**[2*]	0.657	0.126	0.053	0.170	0.034	0.093	0.059	0.287	0.205	0.278	0.301	0.009	0.372	0.032	0.532	Unadjusted

	>0.999	>0.999	0.639	>0.999	0.449	0.928	0.651	>0.999	>0.999	>0.999	>0.999	0.133	>0.999	0.449	>0.999	Adjusted
**ED Visits[2*]**	0.605	0.576	0.023	0.114	0.009	0.699	0.186	0.268	0.055	0.025	0.120	0.003	0.321	0.186	0.009	Unadjusted
	>0.999	>0.999	0.272	>0.999	0.120	>0.999	>0.999	>0.999	0.549	0.280	>0.999	0.051	>0.999	>0.999	0.120	Adjusted

P-values are pooled using five imputed datasets and either a [1]Chi-square test, [2]Fisher exact test, or [3]Kruskal-wallis rank sum test
